# Experimental Evolution of an Oncolytic Vesicular Stomatitis Virus with Increased Selectivity for p53-Deficient Cells

**DOI:** 10.1371/journal.pone.0102365

**Published:** 2014-07-10

**Authors:** Raquel Garijo, Pablo Hernández-Alonso, Carmen Rivas, Jean-Simon Diallo, Rafael Sanjuán

**Affiliations:** 1 Instituto Cavanilles de Biodiversidad y Biologia Evolutiva, Universidad de Valencia, Valencia, Spain; 2 Center for Innovative Cancer Research, Ottawa Hospital Research Institute, Ottawa, ON, Canada; 3 Departamento de Biología Molecular y Celular, Centro Nacional de Biotecnología, Madrid, Spain; 4 Centro de Investigación en Medicina Molecular (CIMUS) and Instituto de Investigaciones Sanitarias (IDIS), Universidade de Santiago de Compostela, Santiago de Compostela, Spain; 5 Department of Genetics, Universidad de Valencia, Valencia, Spain; University Hospital of Navarra, Spain

## Abstract

Experimental evolution has been used for various biotechnological applications including protein and microbial cell engineering, but less commonly in the field of oncolytic virotherapy. Here, we sought to adapt a rapidly evolving RNA virus to cells deficient for the tumor suppressor gene p53, a hallmark of cancer cells. To achieve this goal, we established four independent evolution lines of the vesicular stomatitis virus (VSV) in p53-knockout mouse embryonic fibroblasts (p53−/− MEFs) under conditions favoring the action of natural selection. We found that some evolved viruses showed increased fitness and cytotoxicity in p53−/− cells but not in isogenic p53+/+ cells, indicating gene-specific adaptation. However, full-length sequencing revealed no obvious or previously described genetic changes associated with oncolytic activity. Half-maximal effective dose (EC_50_) assays in mouse p53-positive colon cancer (CT26) and p53-deficient breast cancer (4T1) cells indicated that the evolved viruses were more effective against 4T1 cells than the parental virus or a reference oncolytic VSV (MΔ51), but showed no increased efficacy against CT26 cells. In vivo assays using 4T1 syngeneic tumor models showed that one of the evolved lines significantly delayed tumor growth compared to mice treated with the parental virus or untreated controls, and was able to induce transient tumor suppression. Our results show that RNA viruses can be specifically adapted typical cancer features such as p53 inactivation, and illustrate the usefulness of experimental evolution for oncolytic virotherapy.

## Introduction

Experimental evolution is routinely used to test evolutionary hypotheses under controlled laboratory conditions [1], [2] and in several applied research fields in which natural selection is used to direct specific traits towards pre-defined goals [3]. Directed evolution has allowed researchers to produce proteins with novel or enhanced functions [4], to modify microbial cells for biotechnological applications [5], or even to improve software and develop controllers for autonomous robots [6]. In the field of virology, the classical procedures for creating live attenuated vaccines include serial transfers in non-human hosts under permissive conditions that tend to reduce viral fitness in humans, as well as plaque-to-plaque transfers that allow for the accumulation of deleterious mutations by random genetic drift [7]. Experimental evolution has also been used for predicting the emergence of drug resistance in viruses [8].

Oncolytic virotherapy is an anti-cancer treatment strategy that relies on the ability of viruses to induce selective killing of tumor cells. Currently, there are approximately 100 ongoing or finished phase I, II or III clinical trials involving a plethora of viruses, including adenoviruses, herpes simplex virus, vaccinia virus, parvoviruses, coxsackievirus, poliovirus, retroviruses, reoviruses, measles virus, Newcastle disease virus, or vesicular stomatitis virus (VSV) [9]. Clearly, the dominant approach in the field is to use genetic engineering to make viruses more selective, potent, and safer anti-cancer agents by deleting virulence genes, changing viral envelope proteins to reset viral tropism, and using viruses as vectors of “suicide” genes that are selectively expressed in cancer cells, or of genes that increase susceptibility to radiation and chemotherapy, among other strategies [9]–[12]. However, the rational design of new oncolytic viruses is limited by our incomplete understanding of the complex, extremely diverse, and evolving nature of virus-host interactions. In addition, tumor cells have widely varying properties depending on the cancer type and patient, further complicating this approach [13].

One key aspect of viruses is that, as opposed to conventional therapeutic agents, they are self-replicating and mutating entities and, therefore, are naturally amenable to evolutionary optimization. Therefore, directed evolution should provide a useful complementary approach to genetic engineering for creating new oncolytic viruses or improving the performance of existing ones. However, this approach has been seldom applied to oncolytic virotherapy, albeit with a few notable exceptions [14]–[19]. In one study, pools of adenoviruses from various serotypes were passaged in human colon cancer cells, leading to the isolation of a recombinant virus (ColoAd1) showing improved oncolytic properties relative to the marketed strain [15]. The production of new oncolytic adenoviruses has been enhanced by chemical mutagenesis [19] or using low-fidelity polymerases to replicate the viral genome [16], followed by serial passaging in target cancer cells.

RNA viruses are also suitable for oncolytic virotherapy, and are ideal systems for experimental evolution. Their high rates of spontaneous mutation [20] and often elevated titers allow selection to operate very efficiently, leading to the deterministic evolution of fitness-related traits in the laboratory [21]. Also, their small and compact genomes limit the number of alternative mutations that can be selectively favored in a given environment [22] and, as a result, the same substitutions often appear repeatedly in independently evolving lines (parallel evolution) [23]–[26], facilitating the analysis of the genetic basis of adaptation. Interestingly, selectively advantageous substitutions in one environment tend to become costly in alternate environments (antagonistic pleiotropy), thus producing fitness tradeoffs that favor specialization in a particular host [24], [27]–[29]. These findings strongly suggest that experimental evolution should provide a useful tool for obtaining RNA viruses with increased selectively for tumor cells.

VSV is a prototypic, non-segmented, negative-stranded RNA virus of the family *Rhabdoviridae* and shows some natural selectivity for tumor cells [30]. We hypothesized that it should be possible to increase VSV selectivity for tumor cells by adapting the virus to cells in which the tumor suppressor gene p53 has been inactivated. Since many cancers are p53-defective, viral adaptation to this particular trait may have broad applicability. This approach also allowed us to directly test for fitness tradeoffs associated with adaptation to p53−/− cells by assaying the evolved viruses in isogenic cells with normal p53 function. We found that, after 40 serial passages in p53-knockout mouse embryonic fibroblasts (MEFs), VSV exhibited significantly increased fitness and cytotoxicity in these cells, but that these changes tended to be non-adaptive in normal MEFs, therefore indicating increased selectivity for p53-deficient cells. However, full-length sequencing did not reveal simple molecular signatures underlying this phenotype. Finally, we also demonstrate p53-dependent oncolytic activity in tumor cell cultures and in vivo using mouse 4T1 breast and CT-26 colon cancer models.

## Methods

### Virus and cell culturing

Viral infectious particles were recovered from the VSV cDNA clone originally created by Whelan et al. [31] by transfecting BHK-21 cells as previously described [32]. This virus was used as the founder of the evolution experiments, and was designated as WT. Primary MEFs derived from wild type and p53−/− C57BL6 mice were isolated as previously described [33], cultured in Dulbeco’s modified Eagle’s Medium (DMEM) (Invitrogen) with 10% fetal bovine serum (FBS) (Invitrogen), and passaged upon confluence. p53−/− MEFs were discarded after passage 14 and replaced with a fresh stock to avoid selection of additional mutations. Primary, isogenic, p53+/+ MEFs used in viral fitness and cell viability assays were also kept at a low passage number. 4T1 and CT-26 cells were obtained from the ATCC, cultured in DMEM with 10% FBS and passaged upon confluence. All cells were cultured at 37°C under 95% humidity, 5% CO_2_, and atmospheric O_2_ levels. Viruses were titrated in BHK-21 cells for convenience. In a pilot experiment, we determined that the plating efficiencies of the WT in p53−/− and p53+/+ MEFs relative to BHK-21 cells were 0.53±0.04 and 0.60±0.14, respectively. Viral titers were subsequently corrected by taking plating efficiency into account.

### Experimental evolution

Monolayers containing 10^5^ cells were inoculated with 5×10^3^ plaque forming units (pfu) of VSV (multiplicity of infection, MOI = 0.05 pfu/cell) and incubated for 24 hours post inoculation (hpi), the time required to reach a titer plateau of approximately 2×10^7^ pfu/mL, as determined in preliminary growth curves. The supernatants were titrated after each passage and conveniently diluted to infect fresh cells with a constant number of pfu during the course of the experiment. Assuming a yield of approximately 1000 pfu/cell, the estimated number of viral generations (i.e. infection cycles) per passage was ln(2×10^7^/5/10^3^)/ln 10^3^ = 1.2 [34].

### Viral fitness

Monolayers containing 10^4^ cells were inoculated with approximately 10^3^ pfu of the assayed virus (evolved or WT) and a common competitor which was isogenic to the WT but carried the single-nucleotide replacement A3853C in the plus-strand genome, which confers resistance to a monoclonal antibody but is otherwise selectively neutral [32]. The proportion of each competitor at inoculation and at 24 hpi was determined by titrating the mix in the presence and in the absence of antibody. Fitness relative to the WT was calculated as (R_24_/R_0_)_L_/(R_24_/R_0_)_WT_, where R denotes the titer ratio of the assayed virus to the common competitor at inoculation (0) and 24 hpi (24) for the evolved (L) and WT viruses. Four independent fitness assays were performed for each virus. The plating efficiencies of the evolved lines in p53−/− and p53+/+ MEFs relative to BHK-21 cells were not significantly different from those of the WT (t-test: *P*>0.05).

### Viral cytotoxicity

Cell suspensions containing 10^4^ cells/well were inoculated at MOI>1 pfu/cell, such that most cells became infected. Cell viability was quantified at 18 hpi by adding 20 µL of a 20∶1 (volume) MTS/PMS mix and measuring OD_490_ in a Multiskan FC plate reader (Thermo Scientific). The MOI and incubation time were chosen to provide an estimate of the toxic effect of the virus after a single infection cycle independent of the growth rate of the virus. The fraction of live cells was calculated as V = (OD_i_–OD_0_)/(OD_u_–OD_0_), where sub-indexes i, u, and 0 refer to infected wells, uninfected wells and the blank, respectively. Five independent cytotoxicity measurements were taken for each virus.

### Half-maximal effective dose (EC_50_)

Cell suspensions in 96-well plates (10^4^ cells/well) were inoculated at increasing MOI as indicated and cell viability was quantified by adding Alamar Blue (20 µg/mL final concentration) at 48 hpi, incubating for 2 h, and measuring fluorescence at 590 nm with a 530–560 nm excitation wavelength. The fraction of live cells (V) was calculated as above, and the EC_50_ was estimated by fitting the following model to the data using non-linear least-squares regression: 
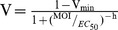
, where V_min_ is the minimal viability value, h measures the maximal rate of viability loss, and MOI is the viral dose (pfu/cell).

### Sequencing

Viral RNA was extracted from infected supernatants using the NucleoSpin RNA Virus kit (Macherey-Nagel), reverse-transcribed with AccuScript (Agilent Technologies), and used for PCR amplification. The genome was amplified using four overlapping PCR fragments (primer sequences available upon request), which were column-purified and used for Sanger sequencing. Chromatograms were analyzed using the Staden software (http://staden.sourceforge.net).

### Western blotting

Confluent 60 mm dishes containing CT-26, 4T1 or Vero cells were exposed to UV irradiation at 4 J/m^2^ for 0, 2, 5 and 10 s, and 16 h after irradiation cells were washed twice with ice-cold Dulbecco’s PBS 1×(PBS), harvested and lysed in a hypotonic buffer (10 mM Tris-HCl pH 7.5, 1.5 mM MgCl_2_, 10 mM KCl, 2 mM DTT, 1 mM Pefabloc, 2 mM sodium vanadate, 4 µg/mL pepstatin, 4 µg/mL leupeptin, and 4 µg/mL aprotinin) for 30 min at 4°C, and centrifuged at 13,000 g for 10 min at 4°C to remove debris. Equivalent amounts of protein were separated by SDS-PAGE and wet-electrotransferred onto PDVF membranes (Roche). Membranes were blocked for 1 h at room temperature with blocking buffer (PBS, 0.1% Tween 20, and 5% nonfat dry milk) and proteins were detected with antibodies against p21 (sc-397, Santa Cruz), and p53 (sc-6243, Santa Cruz). Primary antibodies were diluted 1∶200 in dilution buffer (PBS, 0.1% Tween 20, and 5% nonfat dry milk) and blots were incubated overnight, followed by 1 h incubation with horseradish peroxidase (HRP)-conjugated secondary anti-rabbit antibody (Santa Cruz) (1∶10,000). Peroxidase activity was revealed by enhanced chemiluminescence (Pierce). GAPDH was used as loading control.

### Mouse infections

Virions were concentrated by ultracentrifugation, purified on 5–50% Optiprep (Sigma) gradient and titrated prior to in vivo assays. Six week-old female Balb/c mice were acclimatized for 2–4 days and inoculated subcutaneously in one flank with 3×10^5^ cells resuspended in 100 µL PBS. When tumors reached an estimated size of 220 mm^3^ (8 days for 4T1 and 11 days for CT-26), animals were injected intratumorally with 50 µL of the purified virion suspension containing 10^8^ pfu. A second dose was administered to 4T1 tumors 7 days later. Mice were evaluated every 2–3 days for signs of disease (weight loss, piloerection, anorexia, abnormal behavior). Tumor size was estimated by taking two orthogonal measures using an electronic caliper, and tumor volume was calculated as = (length×width^2^)/2. Animals were euthanized if tumor volumes exceeded 1700 mm^3^ or were highly ulcerated. To test for differences in tumor growth among treatments (WT, L3 and mock-infected controls), we performed a one-way ANOVA in which time was a covariate. These assays were carried out in strict accordance with the “Guide to the Care and Use of Experimental Animals”, as published by the Canadian Council on Animal Care, the provincial legislation entitled “The Animals for Research Act of Province of Ontario” and approved by the Ethics Committee of the University of Ottawa (protocol number ME-222).

## Results

**Figure 1:**
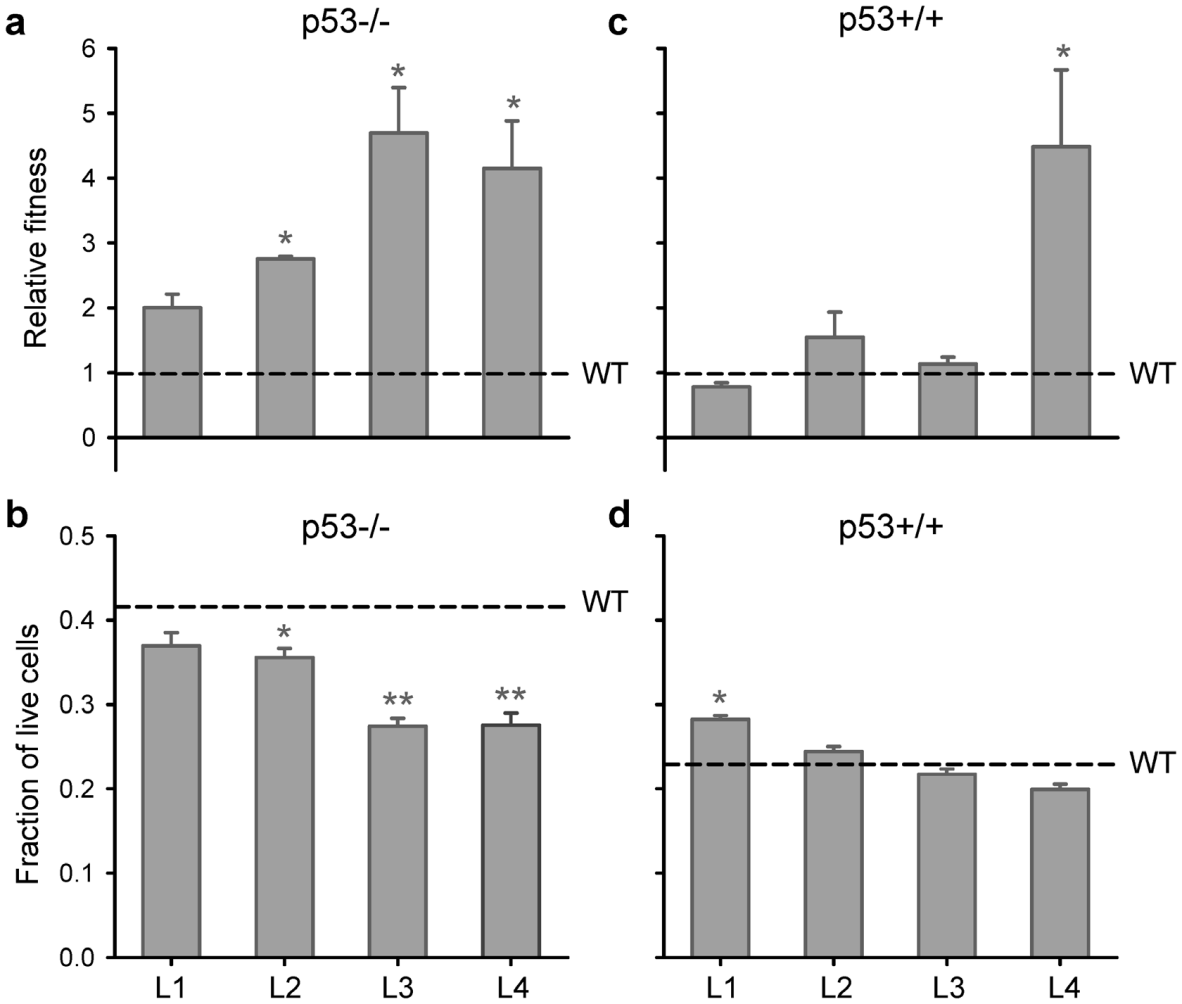
Evolution of viral fitness and cytotoxicity in MEFs. VSV was evolved in p53−/− MEFs for 40 serial passages (MOI = 0.05) and, for each evolved lineage (L1–L4), fitness and cytotoxicity were assayed in p53−/− MEFs (a, b) as well as in isogenic p53+/+ MEFs (c, d). Fitness was determined in four independent competition assays (24 hpi) at a 1∶1 input ratio against a common competitor isogenic to the WT. Cytotoxicity was calculated as the fraction of live cells at 18 hpi relative to uninfected cultures using the MTS assay. For each line, changes in viral fitness and cytotoxicity were evaluated using a one-sample t-test against the WT value (*: *P*<0.05). The horizontal dashed lines indicate WT values, and error bars indicate the SEM.

VSV recovered from an infectious cDNA clone (parental virus, WT) was passaged 40 times in MEFs from p53−/− mice in four replicate lines (L1–L4), which is the equivalent of approximately 50 generations (i.e. infection cycles) of viral evolution per line. For line L3, we observed a significant increase in titer over time (Spearman *ρ* = 0.421, *P* = 0.008), the titer at passage 40 being 3.4 times higher than at the start of the experiment (1.5×10^8^ versus 4.4×10^7^ pfu/mL). To more accurately test if the fitness of the virus had increased in p53−/− MEFs, we competed each evolved line against a common, phenotypically marked, competitor isogenic to the WT virus. This revealed that 3/4 lines (L2–L4) showed significantly higher fitness than the WT (t-test: *P*<0.05), with L3 showing the highest relative fitness (4.7±0.7; Figure 1a). Since previous work with VSV and other viruses has shown that fitness typically correlates with cytotoxicity [35], [36], we expected that that the evolved lines should also show increased ability to kill p53−/− MEFs. To test this, we measured changes in cell viability after one infection cycle using the MTS assay for NADPH flux associated with metabolic activity. This confirmed that the three lines experiencing a fitness increase in p53−/− MEFs were also more cytotoxic than the WT, and that L3 and L4 induced the highest cell death levels (Figure 1b).

To ascertain whether the observed changes were specific to p53-deficient cells, we performed fitness and cytotoxicity assays in isogenic p53+/+ primary MEFs (Figure 1c, d). We found that lines L1, L2, and L3 did not significantly increase their fitness in p53+/+ cells (t-test: *P*>0.3). For L2 and L3, >90% of the fitness gain was lost in p53+/+ cells, indicating that adaptation was mostly gene-specific. In contrast, L4 also showed significantly increased fitness in p53+/+ cells, indicating adaptation to MEFs or to laboratory cell culturing conditions for this line. MTS assays in p53+/+ MEFS revealed that L2, L3 and L4 showed similar toxicity as the WT, whereas L1 was slightly less toxic (Figure 1d). From these results, we conclude that lines L2 and L3 were specifically adapted to p53−/− cells, L1 showed no significant adaptation to p53−/− cells and a slight loss of virulence in p53+/+ cells, and L4 showed significant but non p53-specific adaptation.

**Figure 2:**
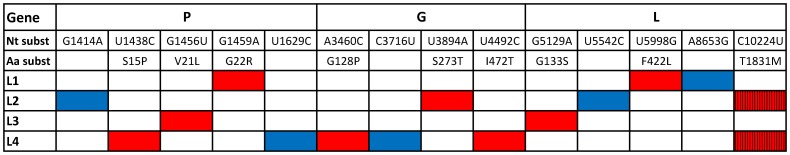
Genetic changes found in the four evolved lines. For each gene, the nucleotide and amino acid substitutions are shown. Blue and red squares indicate synonymous and non-synonymous substitutions, respectively. The C10224U substitution is in hatchet to indicate that this position was polymorphic.

Nearly full-length sequencing (>99% of the genome, bases 60 to 11,100) of the four evolved lines revealed that, in total, 13 different substitutions were fixed and one more was polymorphic at passage 40, with 9/14 substitutions leading to amino acid replacements (Figure 2). Most mutations occurred in genes P and G, reproducing the higher natural variability of these genes [37], [38]. Despite extensive parallel evolution has been reported in previous experimental evolution studies with VSV [23]–[26], we found only one repeated substitution (C10224U), which appeared as a polymorphism in lines L2 and L4. Therefore, there were no clear candidate mutations to confer specific adaptation to p53−/− MEFs, and we thus performed subsequent experiments with viruses derived from passage 40 instead of engineering single-point mutants.

**Figure 3:**
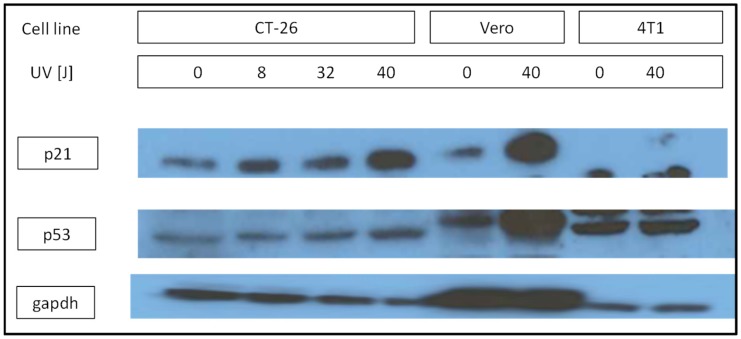
Western blot analysis of p53 expression and function. CT-26, Vero or 4T1 cells were irradiated with UV at the indicated doses to induce DNA damage and tested 16 h post-irradiation for expression of p53, p21, and the constitutively expressed GAPDH. The Western blot shows that p53 protein is expressed in all cells but is functional only in CT-26 and Vero cells, as judged by p21 levels.

**Figure 4:**
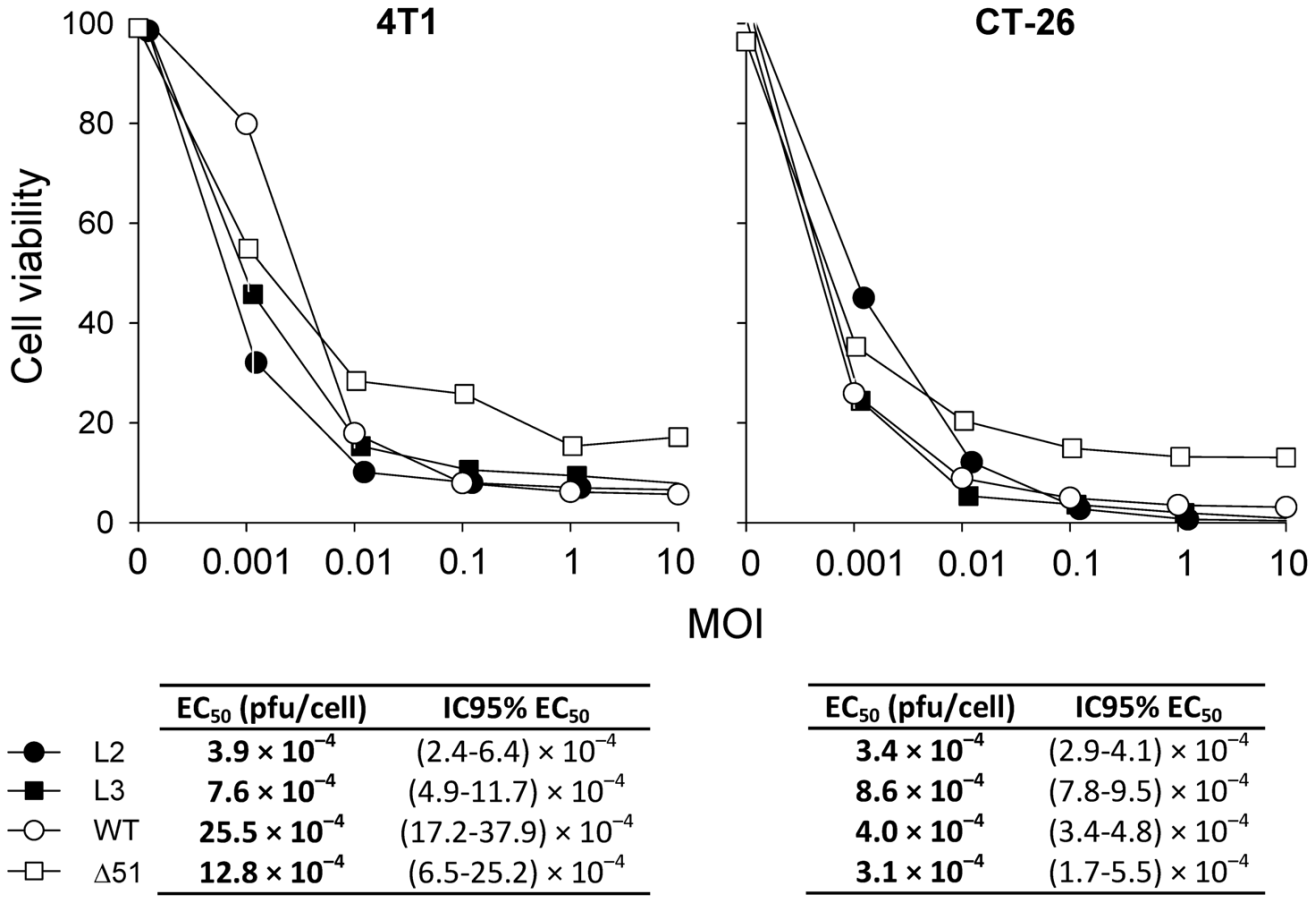
Half-maximal effective dose (EC_50_) of the evolved VSV lines L2 and L3, VSV WT and the VSV mutant MΔ51 in 4T1 and CT-26 cells. Cells were inoculated at the indicated MOI and viability was measured after 48 h using the Alamar Blue assay. EC_50_ values and 95% confidence intervals were inferred from non-linear least-squares regression of cell viability against the MOI, as detailed in the Methods section.

We selected the L2 and L3 lines to characterize their oncolytic activity in 4T1 breast and CT-26 colon cancer cells from Balb/c mice, and we included the well-characterized oncolytic VSV MΔ51 mutant as a reference [39], [40]. We chose these two cell types because 4T1 cells do not express a functional p53 protein, whereas CT-26 cells showed apparently normal p53 function, as revealed by Western blotting of p53 and the downstream p53-activated protein p21 (Figure 3). Cells were infected with an increasing MOI of each virus, assayed for viability at 48 hpi using the Alamar blue oxidation-reduction test, and the EC_50_ was estimated by non-linear least-squares regression (Figure 4). L2 and L3 were more effective against 4T1 cells than the WT virus, as indicated by their significant decrease in EC_50_. In contrast, the L3 and WT viruses were similarly active against p53-positive CT-26 cells, whereas L2 showed a two to threefold increase in EC_50_ in these cells. The VSV MΔ51 mutant was attenuated in both 4T1 and CT-26 cells as judged by the higher cell viability at increasing MOI compared with L2, L3, or the WT.

**Figure 5:**
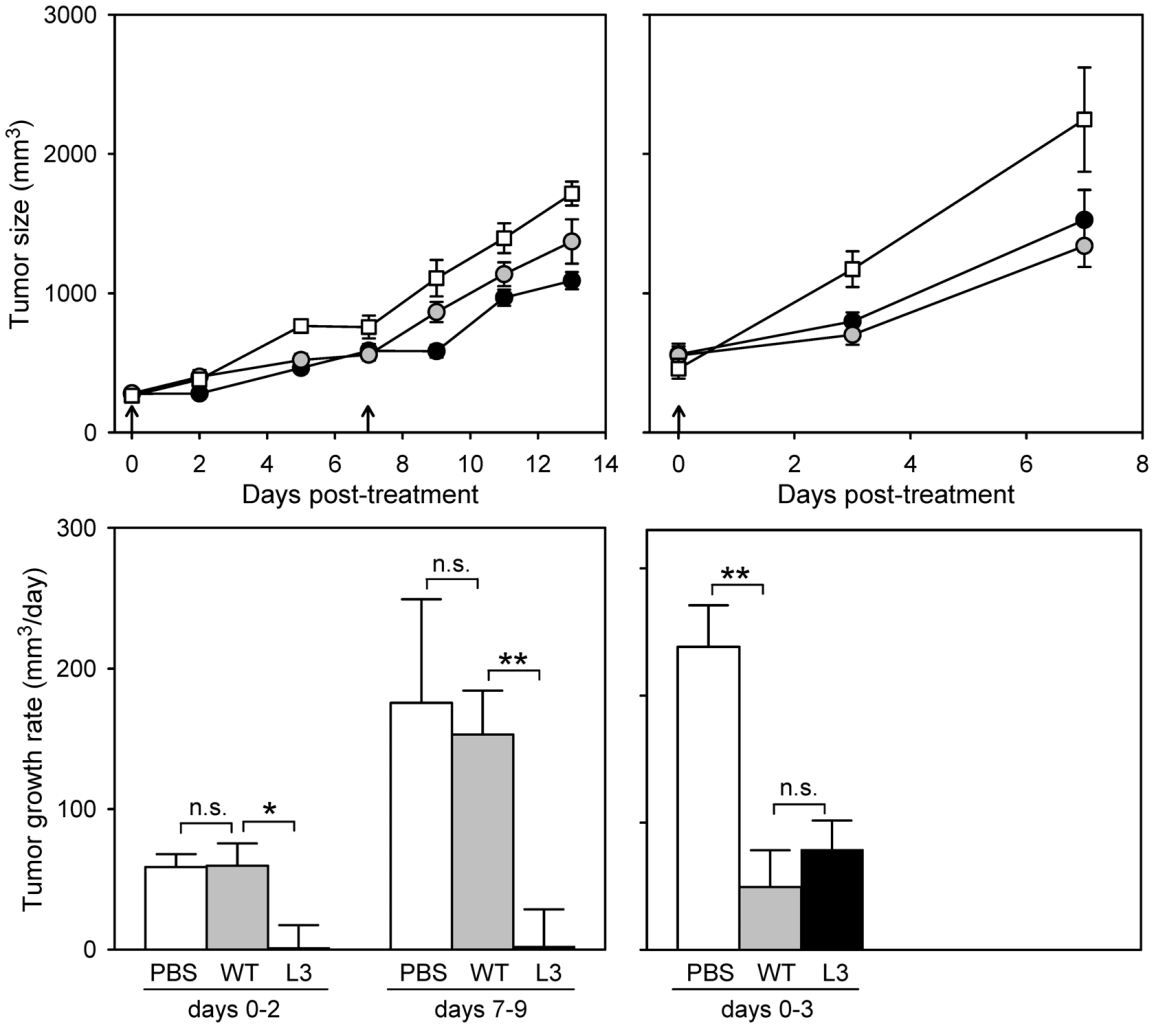
Effect of VSV L3 and WT on the growth of syngeneic Balb/c 4T1 and CT-26 tumors. Viruses were injected intratumorally 8 days (4T1) and 11 days (CT-26) after engraftment, and a second dose was administered 7 days later to 4T1-bearing mice. The experiment was terminated based on end-point criteria on days 14 (4T1) and 7 (CT-26). Top panels (a, c) show tumor growth throughout the course of the experiment for mice treated with the VSV L3 (black circles), VSV WT (grey circles) and the mock-inoculated control group (white squares). Bottom panels (b, d) indicate the tumor growth rate calculated between each viral dose and the first subsequent tumor measurement. The tumor growth rates of each group were compared using t-tests (**: *P*<0.01; *: *P*<0.05; n.s.: *P*>0.05). Error bars indicate the SEM.

Based on the results obtained in MEFs, 4T1 and CT-26 cells, we chose L3 for further evaluation in syngeneic tumor grafts. Twenty-five mice were engrafted with 4T1 cells and, after 8 days, 10^8^ pfu of L3 or the WT were injected intratumorally in each of 10 mice (day 0) and re-injected after one week (day 7). A control group of five mice was mock-inoculated with saline buffer (PBS). Of the five animals treated with the L3 virus, two died at days 7 and 9 from non-tumor related causes. The experiment was terminated on day 13, when 8/23 animals reached endpoint (4/5 untreated, 3/10 treated with the WT, and 1/8 treated with L3). Over the entire course of the experiment, there were significant differences in tumor growth among treatment groups (one-way ANOVA, *P*<0.001), with L3 significantly delaying tumor progression compared with either untreated mice (*P*<0.001) or those treated with the WT virus (*P* = 0.002; Figure 5a). Interestingly, strong differences in tumor growth were observed in the first tumor measurements after each dose (days 2 and 9). Tumor growth was minimal during the two days following the first dose of L3 (1.3±16.1 mm^3^/day) compared with untreated mice (58.7±9.1 mm^3^/day) or mice treated with the WT virus (59.7±15.8 mm^3^/day; Figure 5b). A similar pattern was reproduced during the two days following the second dose (days 7–9), with the L3 virus effectively controlling tumor growth in the short-term, as opposed to the WT virus.

To test whether the increased oncolytic activity of L3 may also be p53-dependent in vivo, we inoculated 24 mice with CT-26 cells and, after 11 days, we applied the same treatment regime as above to 10 mice for each virus type, with four mock-treated controls. The experiment was terminated on day 7 post-treatment because 9/24 animals reached the endpoint criteria. Compared with untreated controls, tumor growth was delayed in mice treated with either the WT (one-way ANOVA: *P* = 0.004) or L3 viruses (*P = *0.041), but there were no differences between the L3 and WT groups (*P* = 0.332; Figure 5c). Focusing on the first measurement following viral inoculation, tumor growth was faster in the controls (238±32 mm^3^/day) than in mice treated with the WT (49.4±28.9 mm^3^/day) or L3 (78.8±22.9 mm^3^/day), but again there were no differences between the two viruses (*P* = 0.436; Figure 5d). Therefore, L3 did not show higher oncolytic activity than the WT in CT-26 tumors.

## Discussion

VSV provides a flexible platform for the design of oncolytic viruses. Nearly all of the approximately 30 different oncolytic VSVs reported in the literature have been produced by genetic engineering, such as introduction of specific mutations in the M and G proteins, generation of pseudotyped viruses expressing the surface protein of other RNA viruses, insertion of microRNAs, or insertion of genes encoding tumor suppressor (p53), suicide (cytidine deaminase, timydine kinase) or immunomodularory (β-interferon, interleukins) proteins [41]. In contrast, very few studies have used evolutionary tools to try to increase the tumor selectivity of VSV. In one such study, an engineered pseudotyped VSV encoding a single-chain antibody against the Her2/neu receptor (ErbB2) was found to yield low titer in target mammary cancer cells expressing ErbB2, and directed evolution was then used to improve viral fitness in these cells [14]. In another study, VSV was serially passaged in human glioblastoma cells to select for more efficient cell attachment, faster replication, and reduced affinity for normal human fibroblasts [17]. The virus rapidly evolved the desired properties and was later shown to be effective against other tumor cell lines [18].

Here, we undertook a more general, experimental evolution approach by serially passaging multiple independent evolution lines in cells deficient for p53 function, a feature shared by many cancers. Based on previous work showing extensive parallel evolution in experimentally evolved VSV [23]–[26], we expected that some substitutions should appear repeatedly after the 40 serial passages. Parallel substitutions are excellent candidates to be selectively advantageous, since it is unlikely that other evolutionary forces such as random genetic drift can lead to the fixation of the same mutations more than once. Therefore, use of multiple lines should help us identify the molecular basis of the observed fitness changes. However, we found that parallel evolution was weak in our lines. The only repeated substitution (C10224U) was polymorphic and was present in one line (L4) showing non-specific adaptation to p53−/− cells. It is possible that, since p53 participates in the innate immune response against VSV [42], p53−/− cells constitute a permissive environment for the virus, thus allowing for the fixation of mutations that are effectively neutral in p53−/− cells but deleterious in cells with normal p53 function by random genetic drift. Still, 3/4 lines showed significantly increased fitness in p53−/− MEFs, indicating the presence of positive selection.

VSV infection leads to secretion of type I interferons (IFNs), which stimulate p53 transcription [42] and p53 post-translational modifications including acetylation, phosphorylation, and SUMOylation [43], [44], promoting apoptosis. As a result, p53−/− cells show partially defective apoptotic antiviral responses, increasing the susceptibility of mice to VSV infection [42]. This is further supported by the finding that a recombinant VSV expressing p53 was highly attenuated in vivo [45]. In turn, the ability of VSV to counteract the innate immune response is mediated mainly by the matrix protein M, which shuts down transcription, inhibits nuclear transport, and interferes with cellular translation [46]. Therefore, mutations in some residues of the M protein such as M51 that partially abolish these functions are tolerated only in cells with crippled IFN pathways, including many tumor cells [39], [40]. Thus, p53−/− cells should offer a permissive environment for the fixation of substitutions in the M protein but, intriguingly, none of the four evolved lines showed any mutation in this gene. The glycoprotein G also has also been located in the nucleus or nuclear membrane [47], and substitutions in several G residues such as E238 have been shown to increase IFN secretion levels and selectivity for tumor cells [48], thus echoing the properties of the M protein. In total, our evolved lines contained three substitutions in the G protein, including S273T in line L2, which maps to the end of the PH domain of the protein in a non-structured region between two α-helices [49], but L3 contained no changes in this gene. Finally, the leader and trailer RNAs may also be implicated in the shutoff of cellular RNA synthesis [50], [51], but the presence of mutations in these regions was not ascertained here.

Most oncolytic viruses including adenoviruses [52], herpex simplex virus [53], and VSV [54] show limited efficacy against 4T1 syngeneic tumors. Treatment of metastatic 4T1 tumors with the oncolytic VSV mutant M51R every two days during two weeks did not achieve tumor regression and increased survival times only modestly [54]. Not surprisingly, similarly modest results were observed here. To date, significant improvement of VSV efficacy against 4T1 tumors has been achieved only in combination with other therapeutic agents, such as for instance by co-infecting tumor cells with vaccinia virus [53], [55] or using drugs such as Sunitinib [56]. To this end, using a more active virus such as VSV L3 may provide additional therapeutic benefit in combination with these agents. Analogously to 4T1, it has been shown that VSV MΔ51 has little effect on CT-26 tumors unless combined with chemical sensitizers that inhibit the IFN response [57]. Interestingly, both the WT and L3 viruses retarded CT-26 tumor growth rates significantly, albeit modestly, compared to untreated controls, supporting the view that viruses in which attenuation is not entirely dependent on the inability block IFN secretion are good candidates to achieve efficient eradication of some IFN-competent tumors. Since experimental evolution is an open-ended process, it may help us identify these and other alternate mechanisms of tumor-specific targeting.
